# 55 Retrospective Chart Review of Use of Self-Adhesive Elastic Wrapping in Patients with Facial Burns

**DOI:** 10.1093/jbcr/iraf019.055

**Published:** 2025-04-01

**Authors:** Renee Warthman, Andria Martinez, Derek Murray, Claudia Islas, Karen Richey, Kevin Foster

**Affiliations:** Diane & Bruce Halle Arizona Burn Center; Diane & Bruce Halle Arizona Burn Center; Diane & Bruce Halle Arizona Burn Center; Diane & Bruce Halle Arizona Burn Center; Diane & Bruce Halle Arizona Burn Center; Diane & Bruce Halle Arizona Burn Center

## Abstract

**Introduction:**

The management of the burn-injured face poses unique challenges for therapy intervention. Standard compression techniques, require an intimate fit and are traditionally implemented after wound closure or autologous grafting when the patient has minimal or no dressings. Garments can impart shear forces during application and may not contour well. We developed a method of self-adhesive facial wrapping to provide custom contoured compression, decrease edema, and help maintain definition of facial features. This wrapping technique can be implemented early, when garments or a facial orthosis cannot be placed. It is used in conjunction with wound care or grafting and accommodates for changes that would preclude other forms of compression. A literature search did not return results describing the use of a self-adhesive wrap on the face. The purpose of this study was to characterize our experience with self-adhesive wrap in adult and pediatric patients with facial burn injuries.

**Methods:**

This was a retrospective chart review of patients treated with self-adhesive elastic facial wrapping at our center from 2019- 2023. Data collected included burn location, TBSA, etiology, demographics, dressing interface, and treatment characteristics including number of applications, duration of use, complications, impact on facial edema, and range of motion of mouth and eyes. Descriptive statistics were calculated.

**Results:**

Thirty subjects were identified in the 5-year period who received self-adhesive elastic wrapping to the face. Mean age was 33 years (range 0.9-82), 56.7% of the subjects were male, mean TBSA was 37.7%, and length of stay was 76.1 days. Initiation of wrap was on average at 21.5 days (range 5-76) after admission, mean duration of treatment was 14.26 days (range 1-113). Some type of insert or oral orthosis was used in conjunction with the wrap for 43%, and of the patients with limitations in oral or ocular closure, all demonstrated improvement after the wrap was applied. There was no relationship between the use of self-adhesive elastic wrap and skin breakdown (p=.743) or graft loss (p=.726). No complications were identified that necessitated discontinuation of the intervention.

**Conclusions:**

The use of self-adhesive elastic wrapping on facial burns helped promote mouth and eye closure, and decreased edema, without discernable complications. Facial compression can be safely initiated early in the phases of wound healing and may help decrease hypertrophic scar formation, subsequently improve function, and decrease the psychosocial impact of burn injury.

**Applicability of Research to Practice:**

The use of this readily available technique may be initiated early in the course of care, to provide early compression when other typical options are not yet appropriate or may be cost prohibitive.

**Funding for the Study:**

N/A

